# Effects of ambient temperature on glucose tolerance and insulin sensitivity test outcomes in normal and obese C57 male mice

**DOI:** 10.14814/phy2.12396

**Published:** 2015-05-19

**Authors:** Anete Dudele, Gitte Marie Rasmussen, David Mayntz, Hans Malte, Sten Lund, Tobias Wang

**Affiliations:** 1 Section for Zoophysiology, Department of Bioscience, Aarhus UniversityAarhus, Denmark; 2 Research and Innovation, VIA University CollegeAarhus, Denmark; 3 Department of Endocrinology and Internal Medicine, Aarhus University HospitalAarhus, Denmark

**Keywords:** Ambient temperature, C57 mice, glucose tolerance test

## Abstract

Mice are commonly used as animal models to study human metabolic diseases, but experiments are typically performed at room temperature, which is far below their thermoneutral zone and is associated with elevated heart rate, food intake, and energy expenditure. We set out to study how ambient temperature affects glucose tolerance and insulin sensitivity in control and obese male mice. Adult male C57BL/6J mice were housed at room temperature (23°C) for 6 weeks and fed either control or high fat diet. They were then fasted for 6 h before glucose or insulin tolerance tests were performed at 15, 20, 25, or 30°C. To ensure that behavioral thermoregulation did not counterbalance the afflicted ambient temperatures, oxygen consumption was determined on mice with the same thermoregulatory opportunities as during the tests. Decreasing ambient temperatures increased oxygen consumption and body mass loss during fasting in both groups. Mice fed high fat diet had improved glucose tolerance at 30°C and increased levels of fasting insulin followed by successive decrease of fasting glucose. However, differences between control and high-fat diet mice were present at all temperatures. Ambient temperature did not affect glucose tolerance in control group and insulin tolerance in either of the groups. Ambient temperature affects glucose metabolism in mice and this effect is phenotype specific.

## Introduction

Mice are a favored animal model to study human diseases as their small size allows for many animals to be kept at relatively low economical costs and because mice are amendable to genetic manipulation (Gordon 2012). Certain mice strains are also popular to study life style diseases because obesity can be readily induced by dietary changes with the associated development of insulin resistance and subsequent type II diabetes (Andrikopoulos et al. 2008; McMurray and Cox 2011; Gordon 2012; Agahi and Murphy 2014). Despite the prevalent use of mice in biomedical research, there is a growing apprehension that captive mice are hypertensive and hyperphagic (e.g., Martin et al. 2010). Much of this concern relates to current housing recommendations stipulating that mice must be kept at 20–26°C (NRC 2011), which is well below their thermoneutral zone (TNZ) of approximately 30–32°C (Gordon 2012; Maloney et al. 2014). The TNZ of mice is substantially higher than humans due to a high overall heat conductance that primarily stems from the high body surface to volume ratio. This implies that mice kept at prescribed temperatures have considerably elevated metabolism with an attending elevation of heart rate (Swoap et al. 2008; Feldmann et al. 2009; Cannon and Nedergaard 2011). The tachycardia is driven by elevated sympathetic tone, whereas the high parasympathetic drive, typical for mammals, dominates within the TNZ (Swoap et al. 2008). However, despite the profound impact of ambient temperature on numerous physiological functions, the influence is often ignored (Karp 2012), and it was recently reported that more than 90% of research articles on mice do not even mention ambient temperature (Maloney et al. 2014).

The glucose tolerance test (GTT) and the insulin tolerance test (ITT) form the basis to characterize glucose homeostasis in humans and experimental animals and are of paramount importance to evaluate the development of diabetes (Andrikopoulos et al. 2008; McGuinness et al. 2009; Bowe et al. 2014). Recently, efforts have been made to standardize procedures for both tests in mice with emphasis on variables such as strain, age, sex, circadian rhythms, fasting duration, route of administration, and dosage (Andrikopoulos et al. 2008; McGuinness et al. 2009; Ayala et al. 2010). However, the influence of ambient temperature has not been considered. This is surprising, because the energetic value of a typical dosage of glucose administered during a GTT (0.0025 g glucose per g mouse) corresponds to almost the entire metabolism of a mouse kept at 30°C for 2 h, whereas a twofold rise in oxygen consumption at 15°C results in a metabolization of the same dosage in only 60 min. Additionally, the increased sympathetic tone that elevates heart rate at low temperatures (Swoap et al. 2008) may alter net glucose uptake by reducing pancreatic insulin secretion and elevating hepatic gluconeogenesis, while simultaneously increasing insulin-independent glucose uptake in muscle and adipose tissue (Nonogaki 2000).

Given the paucity of data on the effects of temperature on blood glucose regulation in mice, we investigate whether acute exposure to temperatures within and below the TNZ affects GTTs and ITTs in a commonly used mice strain (C57BL/6J). To mimic the common laboratory practice where animals are fasted in the laboratory at undefined temperatures, we exposed mice to the experimental temperatures throughout fasting and the metabolic tests. Furthermore, to investigate whether the temperature effects are influenced by body composition and an impaired glucose tolerance, we studied a group of mice rendered obese and diabetic by a high fat diet. As it is recommended to house rodents in groups when possible, we studied temperature effects on animals housed in groups of 5. To control for behavioral thermoregulation that may alleviate cold stress in group-housed animals, oxygen uptake was measured by open respirometry at each experimental temperature while given the same opportunities of behavioral thermoregulation.

## Materials and Methods

### Ethical approval

The experiments were approved by The Danish Animal Experiments Inspectorate, permit number 2012-15-2934-00042.

### Housing and diet

Five-week-old male C57BL/6JBom mice (*n* = 40) were purchased from Taconic (Lille Skensved, Denmark). They were housed in groups of 5 at 23°C and a 12:12-h light:dark cycle with ad libitum access to water at all times. During the first week of acclimation they were fed standard laboratory chow diet and were thereafter divided into two groups with similar weight distributions and given either high-fat diet (HFD, 60% of calories from fat; #D12492; Research Diets Inc., New Brunswick, NJ) or control diet (10% of calories from fat; #D12450B; Research Diets Inc.) for 6 weeks. Food was always freely available, except when fasted prior to the oral GTTs, the intraperitoneal insulin tolerance tests (ITT) or during respirometry. All cages and respirometry chamber were fitted with a handful of paper strands (sizzle nest) for nesting material (Datesand Ltd., Manchester, UK), a polycarbonate mouse igloo (114 mm in diameter and 57 mm high; Brogaarden, Gentofte, Denmark), wooden chew sticks (Aspen S-bricks, Tapvei, Paekna, Estonia), and corn cob bedding (The Andersons Inc., Maumee, OH). Only corn cob bedding was omitted in the respirometry chamber. Twenty mice were subjected to GTTs, and the remaining 20 mice were subjected to both ITTs and respirometry.

### Experimental design

Glucose tolerance tests, ITTs, and open respirometry were carried out at 15, 20, 25, and 30°C and each mouse was subjected to measurements at each of the temperatures with no less than 1 day between ITTs or 1st – 2nd or 3rd – 4th GTT and no less than 6 days between 2nd – 3rd GTT. The order of the temperatures at which the tests were performed was varied systematically. At 7AM on the day of the measurement (approximately 30 min after the lights turned on at the housing facilities) food was removed and the mice were weighed and placed into a clean cage for GTT or ITT, or into the respirometry chamber. Cages and respirometry chambers had the same bedding and enrichment as home cages, except corn cob bedding was omitted during respirometry. For GTT and ITT, the cage was transferred to an illuminated and ventilated climatic chamber set to the desired temperature, and the mice were fasted for 6 h before the test commenced. The mice were returned to the housing facilities with ad libitum access to food after the test.

### Glucose and insulin measurements

Blood for glucose and insulin measurements was collected from an incision at the tip of the tail. Plasma insulin was measured using ultra-sensitive mouse insulin ELISA kit (EIA-3440; DRG International Inc., Springfield, NJ). Blood glucose measurements were performed using Accu-Check Mobile glucometer (Roche, Basel, Switzerland).

### Glucose tolerance tests

Mice were weighed and fasting blood glucose levels were measured immediately prior to the GTT. A 50% glucose solution was given as an oral bolus (2.5 g/kg), and blood glucose was measured at 15, 30, 60, and 120 min after administration.

### Insulin tolerance tests

After weighing the mice, fasting blood glucose levels were measured and were followed by an intraperitoneal injection of insulin (0.05 UI/mL; Actrapid human insulin; Novo Nordisk A/S, Bagsvaerd, Denmark) resulting in a dose of 0.5 UI/kg. Blood glucose was measured at 10, 20, 30, 60, and 120 min after injection.

### Determination of oxygen consumption by open respirometry

To imitate the conditions of GTTs and ITTs, respirometry was performed simultaneously on 5 mice. During the 6 h of measurements food was not available, but water was provided in a bowl. Blood samples were collected to measure fasting glucose and insulin after the respirometry.

A constant flow of outside air, dried through a column of calcium chloride, was maintained through the respirometer and the incurrent flow was measured by a mass flow meter (Sierra Instruments, Monterey, CA). The flow was adjusted so that the excurrent CO_2_ concentration generally was within 0.3–0.7%. A subsample of the excurrent air was dried through a column of calcium chloride and analyzed by oxygen and carbon dioxide analyzers (AEI Technologies Co, Ltd., Pittsburgh, PA) that were calibrated by a Wösthoff gas mixing pumps (Bochum, Germany) before and during the experiments. Rates of oxygen consumption (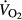) were calculated using the following equation:

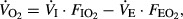
where 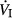 and 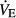 denote the airflow in and out of the chamber, respectively. 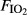 and 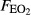, denote the dry fractions of oxygen in the in-flowing and out-flowing air, respectively. As there was no consumption or production of N_2_,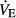 was calculated as:

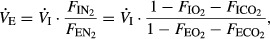
where 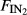 denotes the fraction of N_2_ in the inflowing air, 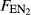 the fraction of N_2_ in the outflowing air, and 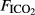 and 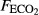 denote the fractions of CO_2_ in the inflowing and outflowing air, respectively.

### Data analysis

Data are expressed as means ± standard error of means (SEM). Data were analyzed using repeated measures analysis of variance (Rep. Meas. ANOVA) with temperature as the repeated factor and diet as the main effect. When diet had a significant effect, Student's *t*-test was performed within the temperature. If temperature or interactions between temperature and diet proved significant Tukey's multiple comparisons post hoc tests were used for pairwise comparison. Results were considered significant at *P* < 0.05. All statistical tests were performed in JMP^®^, Version 10 (SAS Institute Inc., Cary, NC) and Prism 6.0 (GraphPad Software Inc., San Diego, CA). No statistical analyses were performed on measurements of oxygen consumption, as measurements were performed on groups of five animals thus yielding *n* = 2 for each diet at each of the temperatures.

## Results

### Body mass

Initial body mass of the mice used for GTT was 22.4 ± 0.5 g (*n* = 20) and 21.7 ± 0.4 g (*n* = 20) for the mice used for ITT and respirometry. After 6 weeks on the experimental diets, body mass of the GTT mice increased to 29.8 ± 0.7 g on control diet (*n* = 10), and to 35.8 ± 0.7 g on HFD (*n* = 10). During the same period, body mass increased to 27.7 ± 0.7 g (*n* = 10) and 33.4 ± 0.7 g (*n* = 10) on control and HFD, respectively, in the mice used for ITT and respirometry.

**Figure 1:**
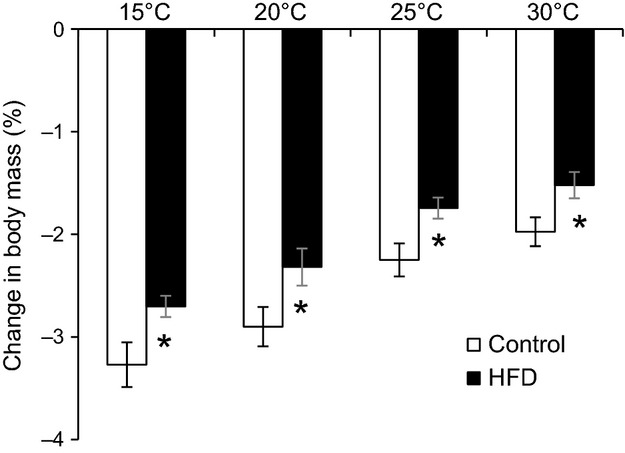
Relative change in body mass after 6 h fast prior to either glucose tolerance test (GTT) and insulin tolerance test (ITT) at different ambient temperatures in C57/6JBom mice fed either control (*n* = 20) or high-fat diet (HFD; *n* = 20) for 6 weeks. Data are presented as overall means ± SEM for both tests. A repeated measures ANOVA revealed significant effects of temperature (*P* < 0.0001) and diet (*P* = 0.001), but no interaction between temperature and diet (*P* = 0.9). * indicates significant effect of diet at the given temperature.

As shown in Figure1, the rate of body mass loss increased with lowered temperatures within both diet groups (Fig.1, Rep. Meas. ANOVA, Temperature: *P* < 0.0001, Diet: *P* = 0.001), whereas no significant interaction was found between diet and temperature (Interaction *P* = 0.9 for both GTTs and ITTs).

### Fasting blood glucose

Fasting blood glucose levels prior to the GTTs (Table1) were not significantly affected by ambient temperature (Rep. Meas. ANOVA, Temperature: *P* = 0.09, Diet: *P* < 0.0001, Diet*Temperature: *P* = 0.06), but were significantly higher in the HFD mice compared to control mice at 20°C (*t*-test, *P* < 0.001), 25°C (*t*-test, *P* < 0.001), and 30°C (*t*-test, *P* < 0.01), but not at 15°C (Table1).

**Table 1:** Fasting blood glucose during the tests

	15°C	20°C	25°C	30°C
Control				
GTT*	10.5 ± 0.6	8.6 ± 0.4	8.8 ± 0.4	8.7 ± 0.3
ITT*†	8.7 ± 0.5	7.4 ± 0.5	7.6 ± 0.3	7.1 ± 0.3
Respirometry*†	9.1 ± 0.4	8.3 ± 0.5	8.7 ± 0.5	7.4 ± 0.4
Pooled*†	9.4 ± 0.3	8.1 ± 0.3	8.4 ± 0.3	7.7 ± 0.2
HFD				
GTT*	11.3 ± 0.5	11.7 ± 0.6	11.9 ± 0.6	10.7 ± 0.5
ITT*†	10.4 ± 0.7	9.2 ± 0.6	9.4 ± 0.4	9.6 ± 0.5
Respirometry*†	12.1 ± 0.7	10.5 ± 0.7	11.1 ± 0.7	9.8 ± 0.5
Pooled*†	11.3 ± 0.4	10.5 ± 0.4	10.8 ± 0.4	10.0 ± 0.3

Fasting blood glucose (mmol/L) during the glucose tolerance test (GTT), the insulin tolerance test (ITT) and the respirometry at the four different ambient temperatures in male C57/6JBom mice after 6 weeks on control or high fat diet (HFD). The overall mean at each temperature is also included (pooled).

Data presented as means ± SEM (*n* = 9–10 in each group, *n*_pooled_ = 29–30).

* indicates significant effect of diet on fasting glucose, whereas

† indicates significant effect of temperature on fasting glucose (Rep. Meas. ANOVA).

Fasting glucose levels obtained prior to ITTs were significantly higher in HFD mice compared to control mice at all temperatures (Table1). Temperature influenced fasting glucose prior to ITTs (Rep. Meas. ANOVA, Diet: *P* < 0.005; Temperature *P* < 0.005; Diet*Temperature *P* = 0.6), however, pairwise comparison revealed that only fasting glucose at 15°C was significantly higher than at 30°C in the control group (Tukey's test, Control, 15°C vs. 30°C *P* < 0.05).

Both diet and temperature had significant effects on fasting glucose after respirometry (Table1, Rep. Meas. ANOVA, Diet: *P* = 0.0005, Temperature: *P* < 0.0005, Diet*Temperature *P* = 0.8) with lowest glucose levels at 30°C and highest at 15°C that were significantly different within both dietary treatment groups (15 vs 30°C, Tukey's test, Control diet: *P* < 0.05; HFD: *P* < 0.005). HFD mice always had significantly higher glucose levels than control mice (*t*-test, 15°C *P* < 0.005, 20°C *P* < 0.05, 25°C *P* < 0.05, 30°C *P* < 0.005).

Pooling all fasting glucose levels measured during three experiments revealed an effect of diet and temperature, but was followed by the effect of the particular experiment (Rep. Meas. ANOVA, Diet *P* < 0.0001, Temperature *P* < 0.0001, Experiment *P* < 0.0005, Diet*temperature *P* = 0.6). Overall this analysis revealed that fasting glucose levels were significantly higher at 15°C in control group that at any other temperature (Tukey's test, Control, 15 vs. 20°C: *P* < 0.005, 15 vs. 25°C: *P* < 0.05, 15 vs. 30°C: *P* < 0.0005), whereas in HFD group fasting glucose at 15°C was only significantly higher than at 30°C (Tukey's test, HFD, 15 vs. 30°C *P* < 0.005).

### Fasting insulin

**Figure 2:**
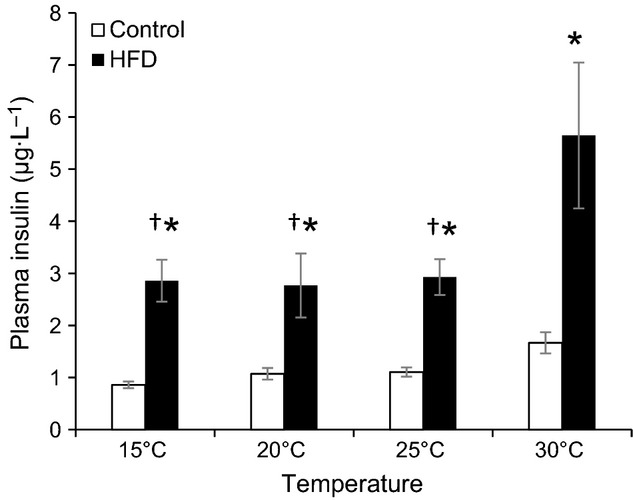
Fasting plasma insulin concentration after 6 h fast during respirometry at different ambient temperatures in C57/6JBom mice fed either control (*n* = 10) or high fat diet (HFD, *n* = 10) for 6 weeks. Data are presented as means ± SEM. A repeated measures ANOVA revealed significant effects of temperature (*P* < 0.005) and diet (*P* < 0.05), but no interaction between temperature and diet (*P* = 0.1). *indicates significant effect of diet at the given temperature, whereas ^†^indicates significant difference from 30°C within the same diet.

**Figure 3:**
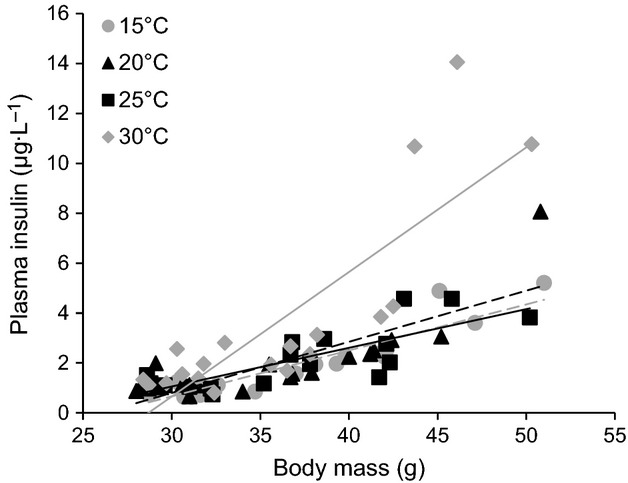
Fasting plasma insulin concentration as a function of body mass after 6 h fast during respirometry at different ambient temperatures in C57/6JBom mice fed either control (*n* = 10) or high fat diet (HFD, *n* = 10) for 6 weeks.

Fasting insulin levels were significantly affected by diet and temperature (Rep. Meas. ANOVA, Temperature: *P* < 0.005, Diet: *P* < 0.05, Diet*Temperature *P* = 0.1) and were always higher in HFD mice compared to control mice (Fig.2) at all four experimental temperatures (*t*-tests, *P* < 0.05 for all four temperatures, respectively). None of the fasting insulin values were significantly different in control mice, but insulin concentration was significantly higher at 30°C in the HFD mice compared to other temperatures (Tukey's test, 15 vs 20°C, 25, and 30°C *P* < 0.0005). Insulin levels correlated well with body mass at 30°C, but this trend was not obvious at the other temperatures (Fig.3).

### Glucose tolerance tests

**Figure 4:**
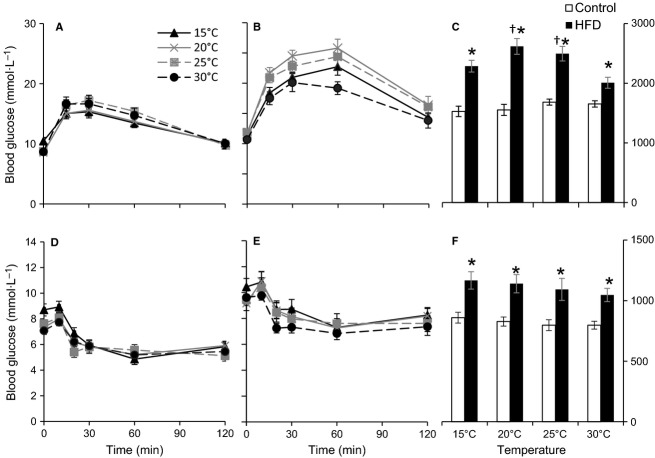
Blood glucose concentrations of control (*n* = 10) (A, D) and high fat diet (HFD, *n* = 10) (B, E) C57/6JBom mice during glucose tolerance test (GTT) (A, B) and insulin tolerance test (ITT) (D, E) after 6 h long fast at different ambient temperatures. Calculated area under the curve (AUC) during GTT (C) and ITT (F). Data are presented as means ± SEM. A repeated measures ANOVA for AUC_GTT_ revealed significant effects of temperature (*P* < 0.05) and diet (*P* < 0.0001), and a significant interaction between temperature and diet (*P* < 0.005). For AUC_ITT_ repeated measures ANOVA revealed no effects of temperature (*P* < 0.2) but a significant effect of diet (*P* < 0.005), and no interaction between temperature and diet (*P* < 0.6). *indicates significant effect of diet at the given temperature, whereas ^†^indicates significant difference from 30°C within the same diet.

Diet significantly influenced glucose levels at all times during the GTTs (Rep. Meas. ANOVA, Diet: *P* < 0.0005 at all times). At the three lower temperatures, HFD mice had significantly higher blood glucose concentrations than control mice throughout the glucose challenge (Fig.4A and B). This was not the case at 30°C, where the initial rise was not significantly higher in HFD mice. The subsequent clearance was slower in the HFD mice, so that blood glucose concentrations remained significantly elevated at 60 and 120 min in the HFD mice compared to the control mice. Both diet and temperature significantly influenced the Area Under the Curve (AUC), and interacted to create different responses within each diet (Fig.4C; Rep. Meas. ANOVA, Temperature: *P* < 0.05, Diet: *P* < 0.0001, Temperature*Diet: *P* < 0.005). However, the AUCs differed significantly between the two diet groups at all temperatures. Within the control mice, temperature did not affect the glucose tolerance and blood glucose concentrations did not differ significantly between temperatures at any time. This was also illustrated by the AUCs, which did not differ significantly between temperatures within the control group (Fig.4C).

Within the HFD mice there were significant effects of the temperature on AUC. The AUC was the lowest at 30°C, followed by AUC at 15°C, and intermediate at 20 and 25°C (Fig.4C). AUC at 30°C was significantly lower than AUCs at 20 and 25°C (Tukey's test, 30 vs 20°C and 25°C *P* < 0.005), but did not differ from AUC at 15°C.

Difference between mean AUC between control and HFD mice at the given temperature (ΔAUC = mean(AUC_HFD_) – mean(AUC_Control_)) was the lowest at 30°C (354.6 units) and threefold higher at 20°C (1064.1 units). At 15 and 25°C ΔAUC was more than two times higher than at 30°C (758.0 and 812.4 units, respectively).

### Insulin tolerance tests

The HFD mice had significantly higher glucose levels than control mice after administration of insulin (Fig.4D and E). AUCs differed significantly between the HFD and the control mice at all temperatures (Fig.4F), but temperature did not influence insulin sensitivity within each diet group (Rep. Meas. ANOVA for AUC, Temperature: *P* = 0.2; Diet: *P* < 0.005, Diet*Temperature *P* = 0.6). The initial rise in glucose levels immediately after handling during the insulin injections was followed by a fall that was more or less maintained until 120 min.

### Oxygen consumption

**Figure 5:**
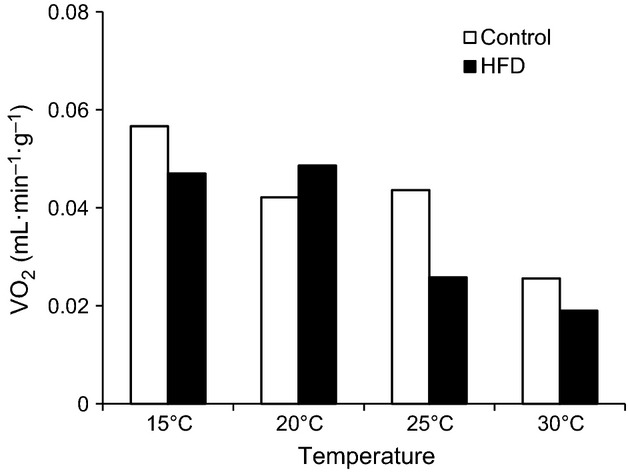
Mass-specific oxygen uptake (VO_2_) after 6 h fast at different ambient temperatures in C57/6JBom mice fed either control (*n* = 10) or high fat diet (HFD; *n* = 10) for 6 weeks. Data are presented as means. Oxygen uptake measured using open respirometry on five mice at a time.

As expected, open respirometry revealed a clear influence of temperature on rate of oxygen consumption of mice (Fig.5). Mice had higher oxygen consumption at the lower temperatures. At all temperatures, oxygen consumption fell substantially within the first hour as the handling-related stress subsided and was steady in the following hours.

## Discussion

Our study shows that environmental temperatures between 15 and 30°C affect blood glucose regulation in mice. While GTTs and ITTs reveal clear differences between control and obese mice at all temperatures, we find that obese and glucose-intolerant HFD mice have increased fasting insulin levels and improved glucose tolerance within TNZ and that control mice have increased fasting glucose levels at 15°C.

Current regulations and guidelines mandate that mice are housed at 20–26°C (NRC 2011), which coincide with room temperatures in most laboratories. These temperatures are well below the murine thermoneutral zone (approx. 30–32°C), and only recently the importance of ambient temperature on experimental outcomes has gained some attention (Lodhi and Semenkovich 2009; Karp 2012; Speakman and Keijer 2013; Maloney et al. 2014). Keeping mice within or close to their TNZ alleviates sympathetic drive (Swoap et al. 2008) that affects glucose handling in a number of ways (Nonogaki 2000). Our results show that mice have a tendency for an overall lower fasting glucose and higher insulin at 30°C (Table1, Figs.3). Plasma insulin levels are also more dependent on body mass at 30°C than at the other temperatures (Fig.3), which may reflect a lowered sympathetic drive within the TNZ allowing for higher insulin secretion (Bloom and Edwards 1975; Gilon and Henquin 2001) that lowers blood glucose. The improved glucose tolerance of HFD mice at 30°C (Fig.4C) also supports this interpretation. Previous studies showed lower plasma glucose concentrations when oral GTTs were performed at 25°C compared to 20°C after a 10 day long acclimation, where the improvements were due to increased insulin production as a result of reduced sympathetic activity (Uchida et al. 2010). Here, we show a similar pattern after short-term exposure to the ambient temperatures (6 h). In fact, HFD mice glucose tolerance is improved at all temperatures when compared with 20°C and best within the TNZ. The oral GTT ΔAUC between control and HFD mice is lowest at 30°C, threefold higher at 20°C and intermediate at other two temperatures (Fig.4C). Thus, performing an oral GTT at 20°C might bring out differences between experimental treatments that would otherwise remain masked at 30°C or even other temperatures, however, the relevance of such findings to human disease modeling must be interpreted with caution (Overton 2010). Interestingly, the observed temperature effects on GTT were phenotype specific and occurred only among HFD mice, whereas there was no effect on control mice. This interaction between the phenotype and ambient temperature may stem from the additional chronic adrenergic drive during obesity (Alvarez et al. 2002; Smith and Minson 2012) and emphasizes that ambient temperature may have divergent effects on mice with different phenotypes.

Similar to a previous study (Uchida et al. 2010), insulin sensitivity assessed by ITT was not affected by temperatures approaching or within TNZ (Fig.4). However, short repeated exposures to high ambient temperatures (41°C) improve glucose tolerance of high-fat-fed rats by improving muscle insulin sensitivity via overexpression of heat-shock proteins (Gupte et al. 2009), but it is unlikely that our maximal experimental temperature (30°C) caused heat stress in the mice (Gordon 2012).

Studies of cold acclimation or acute cold exposure in rodents often report improved diet-induced diabetes, enhanced glucose tolerance in both diabetic and control animals, and reduced levels of insulin, arguably due to increased metabolic demand arising from shivering and non-shivering thermogenesis (Smith and Davidson 1982; Vallerand et al. 1983, 1986). Notably, most of these experiments have been performed on rats exposed to extremely low temperatures (ca. 4°C). We observed a slight non-significant decrease in GTT_AUC_ at 15°C among HFD mice (Fig.4C), but no effects of low temperatures on insulin sensitivity (Fig.4D–F). There were no effects among control mice. However, throughout all three experiments, fasting glucose was generally higher at 15°C than at other temperatures (Table1) for both dietary groups, which may reflect increased liver gluconeogenesis in response to the elevation of noradrenaline during cold exposure (Shiota et al. 1995).

While fasting glucose was affected by ambient temperature in control mice, there was no effect of temperature on their GTT and ITT. Absence of ample differences could be attributed to group housing and availability of nesting material during the measurement that may have sufficiently alleviated thermal stress experienced by mice, thus hindering true effects of cold stress on glucose metabolism (Gaskill et al. 2013). However, previous studies suggest that nesting material and huddling only increases the temperature of the microenvironment by 1°C each (Gordon et al. 1998; Gordon 2004), and we observed a clear elevation of VO_2_ at all temperatures below 30°C in both dietary treatments (Fig.5), consistent with the increased body mass loss during fasting at low temperatures (Fig.1). Thus, we believe that absence of effects on glucose handling must be due to physiological responses and not due to inadequacies in experimental design.

We conclude that environmental temperature, close to the realistic room temperatures in laboratories, affects GTTs in HFD mice, but has no effects on control mice or the ITTs. However, the results may differ among animal models (e.g., altered insulin production or reduced tissue insulin sensitivity) and therefore may lead to different conclusions when conducted at different ambient temperatures. The effects of ambient temperature on glucose metabolism in mice can be unveiled by changing the ambient temperature during the measurements without altering current legislation for husbandry. Due to the differential effects of temperature it is important to further investigate tissue-specific responses to a range of ambient temperatures by using more sensitive and targeted tests.
